# Supplementary material for: Modeling glioblastoma heterogeneity as a dynamic network of cell states
Source: Mol Syst Biol. 2021 Sep 16;17(9):e10105. doi: 10.15252/msb.202010105 (PMC8444284; doi:10.15252/msb.202010105)
Supplement: Supplementary file 5 — Source Data for Figure 3 [file MSB-17-e10105-s001.zip › Figure3A_sourcedata/GSEA_3065/nosigdb_state1.GseaPreranked.1622718409535/DALMO_EXTINCTION_UNPUB.html]

Details for gene set DALMO\_EXTINCTION\_UNPUB[GSEA]

|  || Dataset | state1 |
| Phenotype | NoPhenotypeAvailable |
| Upregulated in class | na\_pos |
| GeneSet | DALMO\_EXTINCTION\_UNPUB |
| Enrichment Score (ES) | 0.741451 |
| Normalized Enrichment Score (NES) | 2.1018155 |
| Nominal p-value | 0.0 |
| FDR q-value | 0.0 |
| FWER p-Value | 0.0 |
Table: GSEA Results Summary

  

Fig 1: Enrichment plot: DALMO\_EXTINCTION\_UNPUB      
 Profile of the Running ES Score & Positions of GeneSet Members on the Rank Ordered List

  

| PROBE | GENE SYMBOL | GENE\_TITLE | RANK IN GENE LIST | RANK METRIC SCORE | RUNNING ES | CORE ENRICHMENT || 1 | CAV1 |  |  | 34 | 0.536 | 0.1197 | Yes |
| 2 | CTSC |  |  | 43 | 0.498 | 0.2335 | Yes |
| 3 | S100A11 |  |  | 51 | 0.475 | 0.3421 | Yes |
| 4 | ARPC1B |  |  | 108 | 0.358 | 0.4188 | Yes |
| 5 | GLIPR1 |  |  | 111 | 0.352 | 0.4996 | Yes |
| 6 | FTL |  |  | 134 | 0.333 | 0.5739 | Yes |
| 7 | CNN2 |  |  | 208 | 0.291 | 0.6334 | Yes |
| 8 | COL1A2 |  |  | 517 | 0.201 | 0.6485 | Yes |
| 9 | NDRG1 |  |  | 765 | 0.163 | 0.6608 | Yes |
| 10 | BACE2 |  |  | 864 | 0.149 | 0.6852 | Yes |
| 11 | THBS1 |  |  | 1118 | 0.121 | 0.6873 | Yes |
| 12 | RAB27A |  |  | 1182 | 0.114 | 0.7071 | Yes |
| 13 | PRSS23 |  |  | 1356 | 0.099 | 0.7123 | Yes |
| 14 | PLAUR |  |  | 1463 | 0.091 | 0.7225 | Yes |
| 15 | SDC1 |  |  | 1482 | 0.090 | 0.7415 | Yes |
| 16 | LOX |  |  | 1825 | 0.068 | 0.7224 | No |
| 17 | P4HA2 |  |  | 1910 | 0.065 | 0.7288 | No |
| 18 | RGS2 |  |  | 1971 | 0.061 | 0.7367 | No |
| 19 | CYP1B1 |  |  | 2236 | 0.050 | 0.7214 | No |
| 20 | RUNX1 |  |  | 3487 | 0.014 | 0.5977 | No |
| 21 | BCL3 |  |  | 3622 | 0.011 | 0.5867 | No |
| 22 | TGFBI |  |  | 3688 | 0.010 | 0.5824 | No |
| 23 | TNFAIP8 |  |  | 3901 | 0.006 | 0.5623 | No |
| 24 | ITGB1 |  |  | 3956 | 0.005 | 0.5580 | No |
| 25 | SYNGR2 |  |  | 4130 | 0.002 | 0.5409 | No |
| 26 | LY96 |  |  | 4335 | -0.002 | 0.5206 | No |
| 27 | CTSB |  |  | 4873 | -0.011 | 0.4685 | No |
| 28 | COL5A1 |  |  | 7642 | -0.067 | 0.2030 | No |
| 29 | NNMT |  |  | 8484 | -0.104 | 0.1416 | No |
Table: GSEA details [plain text format]

  

Fig 2: DALMO\_EXTINCTION\_UNPUB: Random ES distribution      
 Gene set null distribution of ES for **DALMO\_EXTINCTION\_UNPUB**
